# Neurology

**Published:** 1895-10

**Authors:** William C. Krauss, James W. Putnam

**Affiliations:** Professor of nervous diseases in the Medical Department of Niagara University; Professor of nervous diseases, Medical Department of the University of Buffalo


### Progress in Medical Science.

### NEUROLOGY.

CONDUCTED BY WILLIAM C. KRAUSS, M. D.,
Professor of nervous diseases in the Medical Department of Niagara University, and

JAMES W. PUTNAM, M. D.,
Professor of nervous diseases, Medical Department of the University of Buffalo.

### A HUMAN TIMEKEEPER.

DR. L. R. CULBERTSON (*Cincinnati Lancet-Clinic*, August 17, 1895,) narrates the case of a negro who, when asked the time of day, will put his right or left index finger on his temple, think for an instant, and, without looking at a watch or clock, tell the exact time, or, at least, not vary more than ten minutes. On being awakened at night he can tell the time within a minute or two, as was evidenced by the author. When a boy, he was struck on the forehead with a mattock, which left a depression in the frontal bone one-eighth of an inch deep. Although the cicatrix is adherent, he has never had any epileptic attacks, no paralysis or paresis, no loss of mind or memory, was never unconscious nor suffered any ill effects therefrom. He also states that when a boy he would sit and watch the clock a great deal and would walk and count the number of steps and the length of time it took to cover a certain distance. The writer tries to explain this abnormal faculty in one of two ways. First, there may be a small piece of bone pressing on the superior or middle frontal convolution, causing irritation or over-stimulation of the cortical centers presiding over time ; or, secondly, that by practice he has so cultivated the conscious and unconscious cells presiding over time that they are highly developed in this respect. The writer seems more inclined to the latter view, inasmuch as the time center has as yet not been located, and since, by constant stimulation, any one cortical center or group of centers may attain a remarkable development, so, in this case, the noting of time space has developed these centers to a remarkable degree.

---

### TREATMENT OF THE FULGURATING PAINS OF LOCOMOTOR ATAXIA.

BLONDEE (*Revue de Therapeutique*, April, 1895,) describes a novel way of treating these obstinate pains, by the following simple methods : Its *rationale* consists in the elongation of the spinal cord in the canal, without suspension and the danger of luxation that accompanies that method. The patient is told to perform the following exercise each evening before going to sleep : lying flat upon his back on the bed, he should flex his thighs upon the body and the legs upon the thighs, bringing the knees as near as possible to the chin, advancing the head to meet the knees as much as possible. A band is then passed about the neck and beneath the knees, enabling the patient to retain this position for five minutes. It is evident that the cord will be stretched, especially in the region of the dorsal columns of the spinal cord or the diseased portion.

---

### A CONSTANT SIGN IN COMMENCING MENINGITIS.

IN THE July number, 1895, of the *Alienist and Neurologist* is given the following description of a phenomenon observed in the very early stage of meningitis : it consists in the inharmonious movements of the chest and diaphragm. The chest and abdomen must be bared, but not suddenly, or the hyperesthetic skin will take on accidental movements from the action of the air. In the first period of meningitis may be seen irregularity of rhythm and inequality of the amplitude or development of the chest. Another sign is the irregular type of respiration and dissonation of the movements of chest and diaphragm. The respiration is effected by the lower respiratory muscles of the chest. Looking at the umbilical region, instead of the normal elevation, with each inspiration there is either immobility or depression. These movements are not connected with the Cheyne-Stokes type of respiration.

### ACONITINE IN NEURALGIA.

HUNSBERGER (*Therapeutic Gazette*, August 15, 1895,) read a paper on the use of aconitine in neuralgias of various kinds before the Pennsylvania State Medical Association, May 23, 1895, and his experiences are most favorable toward the drug. In trigeminal neuralgias the writer believes that surgical interferences are only palliative measures, the apparent good results noted for a short time after the operation being due to the counter-irritation of cutting down to the nerves. Aconitine in 1-200 grain dose every two hours continued for several days had better effect than all other remedies in the writer's hands.

In intercostal and visceral neuralgia, aconitine seems to have a peculiarly good effect, due, in part, to the fact that it does not irritate the digestive tract.

In neuralgia of the sciatic nerves, aconitine should be given in small doses for two consecutive months, and again for the same period after a cessation of two weeks. The writer has treated successfully fifty-seven cases of neuralgia of the various organs and has never seen any bad symptoms follow or any injurious action of the heart produced, the appetite was never diminished, no constipation, no nausea, no dryness of the tongue or throat, or dull headache follow its use—symptoms that follow the use of all preparations containing morphine, atropine and other drugs.

---

### TESTS FOR EARLY LOCOMOTOR ATAXIA.

FOURNIER, (*Medical Herald*,) in giving the points for early recognition of tabes dorsalis, classifies the diagnostic signs as follows: (1) Westphal's symptom is well known; it consists in the abolition of the pro-rotulian reflex, and is present in two-thirds of the cases. (2) Romberg's sign can be thus appreciated: the eye is an indirect regulator of motion; it helps to correct deviations in walking and maintains the equilibrium. When a patient is suspected of incipient ataxy, it will often suffice to make him close his eyes when in the erect position to verify the diagnosis. In a few instances his body will oscillate, and if the malady is somewhat advanced he will be in danger of falling. (3) The "stairs" symptom. One of the first and most constant symptoms of incipient locomotor ataxy is the difficulty with which the patient will descend stairs. If questioned closely on the subject he will say that at the very outset of his malady he was always afraid of falling when coming up stairs. (4) The manner in which a patient crosses his legs is often significant. In the normal state a man when performing that act lifts one leg simply to the height necessary to pass it over the other, whereas in the affection under consideration he lifts it much higher than necessary, describing a large segment of a circle. (5) Walking at the word of command. The patient seated is told to get up and walk instantly. After rising, he will hesitate as if he wanted to find his equilibrium before setting off. If while in motion he is told to stop short, his body, obeying the impulsation, inclines forward as if about to salute, or, on the contrary, jerks himself backward in order to resist the impulsion forward. (6) The patient is asked to stand on one leg, at first with his eyes open, afterward closed. Although man is not made for this position, yet he can balance himself pretty firmly for a little time. The ataxic will experience a great deal of difficulty, and will instinctively call to aid his other foot, so as not to fall. If his eyes are closed, he will not be able to stand an instant, and if not held would fall heavily to the ground. Such are the symptoms of incipient locomotor ataxy. They will not all be present frequently, but they should be all sought for, in order to avoid an error which might have grave consequences.

---

### NERVE SUTURE.

Sinkler (*Journal of Mental and Nervous Diseases*, June, 1895,) reports a case of injury to the musculo-spiral nerve by a pen knife followed by complete extensor paralysis. Operation three months later by Dr. Keen. The nerve was found to be severed. The end of the nerve was bulbous. The bulb was excised and the extremities united by silk sutures. It was treated for seven months by galvanism before the patient was able to fully extend the fingers and hand.

This case is interesting from two points of view—first, in showing that even after union of the nerve by suture, months of patient work are needed to restore function. The case also illustrated that suture need not be immediate to be effective. This is, of course, well known, as many cases have been reported in which the interval between the injury and the operation was much longer than three months.

---

### REMOVAL OF UTERINE APPENDAGES FOR NERVOUS DISEASE.

This subject was recently discussed (*Boston Medical and Surgical Journal*, March 7, 1895,) by Drs. Knapp, Prince, Homans, Edes and Davenport. The general opinion expressed was that it was extremely doubtful whether disease of the uterus or ovaries ever gave rise to nervous disease without having special symptoms pointing to those organs.

That there was a curative effect in the removal of healthy tubes and ovaries for nervous diseases as claimed by Dr. W. H. Baker was generally denied.

---

### PALMUS.

Landon Carter Gray (*Journal Mental and Nervous Diseases*, May, 1895,) advocates the adoption of this name to designate the French tic convulsive.

He divides it into four types: 1. Facial palmus. 2. General palmus. 3. Acute palmus. 4. General palmus with pseudo-melancholia. The characteristics of palmus are sudden, rapid and shock-like movements.

---

### SPASMODIC TORTICOLLIS WITH CASES.

Richardson and Walton (*American Journal Medical Sciences*, January, 1895,) arrive at the following conclusions:

1. Palliative treatment, whether by drugs, apparatus or electricity, will rarely prove successful in well-established spasmodic torticollis.
2. Massage may prove of value in comparatively recent cases.
3. Resection affords practically the only rational remedy.
4. Operation on the spinal accessory nerve may afford relief, even if other muscles than the sterno-cleido-mastoid are affected; on the other hand, the affection previously limited to the sterno-cleido-mastoid may spread to other muscles in spite of this operation.
5. No fear of disabling paralysis need deter us from recommending operation, as the head can be held erect even after the most extensive resection.
6. The most common combination of spasm is that involving the sterno-mastoid on one side and the posterior rotators on the other, the head being held in the position of sterno-mastoid spasm with the addition of retraction through the greater power of the posterior rotators.
7. It seems advisable in most cases to give preference to the resection of the spinal accessory as the preliminary procedure.

### TRAUMATISM AS A CAUSE OF LOCOMOTOR ATAXIA.

PRINCE (*Journal of Mental and Nervous Diseases*, February, 1895,) reports three alleged cases of ataxia of traumatic origin and reviews forty reported cases. These he arranges in three groups.

Group one, twenty-two cases inadmissible on account of triviality of injury, preëxistence of syphilis, long interval between injury and onset of symptoms, or doubtful diagnosis.

Second, group of six cases, which, while traumatism cannot be excluded, still cannot with safety be admitted as credible evidences of a traumatic etiology.

The third group of twelve cases may reasonably be regarded as the result of traumatism, and even in these twelve cases there is not one in which flaws are not found in their histories. Of all the published alleged cases he only finds four to which no specific objection can be raised. He concludes that, taking all the facts into consideration, it would seem that the current view that locomotor ataxia may be caused by traumatism *per se*, irrespective of a direct lesion of the cord, is not sustained by evidence. It is more probable that, when a sclerosis develops after a traumatism, the subject was already doomed to this condition, the process having already begun, and that the traumatism, at most, accelerated the development of the symptoms and, possibly, of the anatomical process.

